# Burn Wound Infections and Antibiotic Susceptibility Patterns at Pakistan Institute of Medical Sciences, Islamabad, Pakistan

**Published:** 2015-01

**Authors:** Muhammad Saaiq, Shehzad Ahmad, Muhammad Salman Zaib

**Affiliations:** Department of Plastic Surgery and Burn Care Centre, Pakistan Institute of Medical Sciences (PIMS), Islamabad, Pakistan

**Keywords:** Burn, Infection, Antibiotic sensitivity, Pakistan

## Abstract

**BACKGROND:**

Burn wound infections carry considerable mortality and morbidity amongst burn injury victims who have been successfully rescued through the initial resuscitation. This study assessed the prevalent microrganisms causing burn wound infections among hospitalized patients; their susceptibility pattern to commonly used antibiotics; and the frequency of infections with respect to the duration of the burn wounds.

**METHODS:**

This study was carried out at Burn Care Centre, Pakistan Institute of Medical Sciences (PIMS), Islamabad, Pakistan over a period of two years (i.e. from June 2010 to May 2012). The study included all wound-culture-positive patients of either gender and all ages, who had sustained deep burns and underwent definitive management with wound excisions and skin auto-grafting. Patients with negative cultures of the wounds were excluded. Tissue specimens for culture and sensitivity were collected from burn wounds using standard collection techniques and analyzed at microbiological laboratory.

**RESULTS:**

Out of a total of 95 positive microbial growths, 36 were* Pseudomonas aeruginosa* (35.29%) as the most frequent isolate found, followed by 21 *Klebsiella pneumoniae* (20.58%), 19 *Staphylococcus aureaus* (18.62%), 10 *Proteus *(9.80%), 7 *E. coli* (6.86%), 7 *Acinetobacter* (6.86%), and 4 *Candida* (3.92%). A variable antibiotic susceptibility pattern was observed among the grown microbes. Positive cultures were significantly more frequent among patients with over two weeks duration of burn wounds.

**CONCLUSION:**

*P. aeruginosa*, *K. pneumoniae* and *S. aureus* constituted the most common bacterial microbes of burn wounds in our in-patients cases. Positive cultures were more frequent among patients with over two weeks duration of burn wounds. Early excision and skin grafting of deep burns and adherence to infection control measures can help to effectively reduce the burden of these infections.

## INTRODUCTION

Infection in burn wound is still considered as the most important cause of disability and mortality in all ages and in both developed and developing countries.^1^^,^^2^ Despite considerable advances in the overall management of burn injuries, infection and the resultant sepsis continues to be a formidable foe for burn care providers. Approximately 50-75% of mortality amongst burn patients after the initial resuscitation phase, is attributable to various infectious complications.^3^^-^^5^

Burn injury patients are at high risk of infections for a variety of reasons. For instance, the readily available exposed body surface, immunocompromizing effects of burns, invasive diagnostic and therapeutic procedures and prolonged hospital stay. Patient factors such as age, extent of injury, and depth of burns coupled with microbial factors such as the type and number, enzyme/toxin production and motility of organisms are the determinants of invasive infection. Superficial bacterial contamination of the wound can easily advance to invasive infection in these patients. The degree of bacterial wound contamination has a direct correlation with the risk of sepsis.^5^^-^^7^

Factors that are associated with improved outcome of burn injury patients and prevention of infections among them, predominantly include early excisions and grafting of deep burns together with aggressive infection-control measures.^5^^,^^8^ These changes potentially lead to the emergence of antibiotics resistant isolates and treatment failures. In topical burn therapy, silver sulfadiazine (SSD) is considered as the gold standard with antibacterial properties.^9^

Several studies were performed to develop dressings to accelerate the healing process and to decline the bacterial burden in burn wounds. Even medicinal plants were introduced in healing of burned wounds, traditional forms of medicine, especially herbal products, which have been employed for centuries are under scientific investigation to evluate their roles in wound healing.^10^^-^^14^

Blood infections are life-threatening if not diagnosed and treated properly. Antibacterial susceptibility patterns for micro-organisms isolated from the hospitalized patients with infectious diseases are continuously evolving.^15^ Determining the sensitivity of isolated bacteria to antibiotics consumption may help clinician use appropriate antibiotics. Due to unavailability of an appropriate alternative antibiotic for hospitalized patients, it may be life-threatening in some patients.^16^ So the present study was conducted to determine the microbial profile of burn wounds, the antimicrobial susceptibility patterns of the cultured microbes and the frequency of infections with respect to the duration of wound, among our burn injury patients.

## MATERIALS AND METHODS

This study was carried out at Burn Care Centre, Pakistan Institute of Medical Sciences (PIMS), Islamabad over a period of two years (i.e. from June 2010 to May 2012). It included patients of either gender and all ages, who had sustained deep burns and underwent definitive management with wound excisions and skin autografting. Patients with negative culture reports were excluded. Initial assessment was made by history, physical examination and necessary investigations. All patients were initially resuscitated and optimized on standard management outlines. Before undergoing wound excisions, the wounds of hospitalized patients were managed with topical 1% SSD cream with bulky dressings and changed every 24 hour. Any documented infection was managed with systemic antibiotics as dictated by their culture and sensitivity reports.

Convenience sampling technique was used for tissue biopsies for culture and sensitivity and tissue specimens were collected at the time of excisions of the deep burn wounds. The operative procedures undertaken among the patients included complete excision of deep burns and skin auto-grafting with split thickness skin grafts. The patients presenting early had early excisions while those presenting late had delayed excisions and grafting. 

As the study was an observational one and did not involve any new intervention, it was conducted in accordance with the Declaration of Helsinki of 1975, as revised in 2013 and anonymity of the participants was ensured.

Specimens for tissue culture and sensitivity were collected by employing standard collection techniques and analyzed at microbiological laboratory for culture and sensitivity. After inoculation on appropriate culture media, the specimens were incubated for 24 hours at 37ᵒC for obtaining aerobic and anaerobic growths. The microbes were identified by their colonial morphology and characteristic biochemical tests. Antibiotic susceptibility was tested by employing disc diffusion method using standard antibiotic discs.^17^

The data were analysed through SPSS software (Statistical package for social sciences, Version 11, Chicago, IL, USA). Various descriptive statistics were used to calculate frequencies, percentages, means and standard deviation. The numerical data such as age of the patients and the total body surface area (TBSA) affected were expressed as mean±standard deviation while the categorical data such as the organisms cultured were expressed as frequency and percentages. The percentages of various outcome variables were compared by employing Chi-Square test and a *p*-value of less than 0.05 was regarded statistically significant. 

## RESULTS

The tissue specimens of 95 patients yielded microbial growths. Out of these, 53.68% (n=51) were males while 46.31% (n=44) were females. The age of the patients ranged from 1 year to 55 years (mean: 23.95±11.83 years) whereas the TBSA burned ranged from 5% to 40% (mean: 21.53±9.59%). The types of initial burn insults included flame burns in 72 (75.78%) patients, electrical burns in 13 (13.68%) patients, scalds in 9 (9.47%) patients and acid burns in 1 (1.05%) patient. Positive cultures were significantly more frequent among patients with wounds of over weeks duration (84.21%; n=80) than those with less than 2 weeks duration (15.79%; n=15) (*p*=0.001). 

Out of the 95 microbial growths, 92.63% (n=88) of isolates were monobacterial whereas 7.36% (n=7) were multibacterial. *Pseudomonas aeruginosa* (n=36; 35.29%) was the most frequent isolate followed by *Klebsiella pneumoniae* (n=21; 20.58%), *Staphylococcus aureaus* (n=19; 18.62%), *Proteus* (n=10; 9.80%), *E. coli* (n=7; 6.86%), *Acinetobacter* (n=7; 6.86%), and *Candida* (n=4; 3.92%). Among the *S. aureaus* isolates, majority (n=13; 68.42%) were Methicillin resistant (MRSA). The sensitivity pattern among the cultured bacteria is shown in Table 1. 

**Table 1:** Dominant sensitivity pattern of the frequently cultured microbes among the study patients (n=95).

**Antibiotics tested**	***Pseudomoas aeruginosa*** **(n=36)** **No. (%)**	***Klebsiella pneumoniae*** ** (n=21)** **No. (%)**	**Proteus** **(n=10)** **No. (%)**	**MRSA** **(n=13)** **No. (%)**	**MSSA** **(n=6)** **No. (%)**
Piperacillin+Tazobactum	29 (80.55)	17 (80.95)	7 (70.00)	-	-
Imipenem	23 (63.88)	16 (76.19)	10 (100)	-	-
Ciprofloxacin	16 (44.44)	5 (23.80)	-	-	3 (50)
Polymyxin B	13 (36.11)	4 (19.04)	-	-	-
Cefoperazone+Salbactum	11 (30.55)	13 (61.90)	7 (70.00)	-	-
Amikicin	3 (8.33)	13 (61.90)	5 (50.00)	-	-
Levofloxacin	2 (5.55)	14 (66.66)	3 (30.00)	-	-
Ceftazidime	4 (11.11)	5 (23.80)	-	-	-
Vancomycin	-	-	-	13 (100)	6 (100)
Fusidic acid	-	-	-	9 (69.23)	6 (100)
Linezolid	-	-	-	13 (100)	6 (100)
Chloramphenicol	-	-	-	9 (69.23)	4 (66.66)
Cloxacillin	-	-	-	-	5 (88.33)

## DISCUSSION

Wound infection is one of the most feared complications through the course of burn wound management. In fact, in the absence of appropriate care, the burn wound is an ideal culture medium for the colonization and proliferation of all kinds of endogenous and exogenous microbes. The skin that normally serves as a physical barrier against microbes is disrupted, thus making the individual susceptible to microbial invasion. Additionally the underlying vasculature of the skin has also been damaged to variable extent, making it difficult for various components of the immune response to reach the affected site to combat infection. Accordingly, the risk of infection increases proportionately with the size and depth of the burn. The type and quantity of microbes colonizing the burn wound influence the frequency of invasive infections and their clinical severity.^3^^,^^4^

In our study we performed tissue biopsies for culture and sensitivity to determine wound infection. The American Burn Association has recognized the spectrum of infections in burns as wound colonization, wound infection, invasive infection, cellulitis, and necrotizing infection/fasciitis. Wound colonization refers to the presence of low concentrations of bacteria on the surface without invasion or systemic manifestations. Wound infection is associated with bacterial count of >10^5^ bacteria per gram of tissue within the wound or eschar. An invasive infection is associated with a bacterial count of >10^5^ per gram that causes suppurative separation of the eschar or graft loss with involvement of unburned tissue or the presence of systemic sepsis. Cellulitis manifests as erythema, induration, warmth, and tenderness in the tissue surrounding the burn wound while necrotizing infection involves an aggressive invasive infection with involvement of structures below the skin.^5^^,^^18^

*P. aeruginosa* was the most frequent microbial isolate in our patients. Our finding conforms to many published studies which have reported *P. aeruginosa* as the commonest microbe cultured from burn wounds.^15^^,^^16^^,^^19^^-^^22^ In striking contrast to our finding, some published studies have reported *S. aureus* as their predominant microbe of bacterial burn wound infections.^23^^,^^24^ In the context of pseudomonal infections, ceftazidime has long been perceived as an effective antibiotic, however in our study only 11.11% of *P. aeruginosa* isolates were sensitive to it. With this evidence base in mind, we can revisit our policy of empiric antibiotic cover for our burn injury patients with sepsis. 

*Klebsiella *species constituted the second most frequently identified organism in our series, conforming to published studies which have also reported it as the second commonest cultured microbe,^25^ however other studies have reported *Klebsiella *species as the most common microbe in the bacteriologic profiles of their burn wounds.^26^^-^^28^

In our study *S. aureus *was the third most frequently identified organism. Gram-positive bacteria in the depths of sweat glands and hair follicles have been reported to survive the heat of initial burn injury and unless topical antimicrobial agents are applied, these bacteria heavily colonize the wounds within the first 48 h post injury. Measures to prevent and treat infection by these *Staphylococci *are essential for the survival of patients with extensive burns.^3^^,^^22^^,^^25^

In our study methicillin-resistant *S. aureus (*MRSA) constituted an alarmingly high percentage (68.62%) of the *Staphylococcal* isolates. With the exception of one patient, the remainder had been receiving home treatment for over two weeks of dressings only, before presenting to our unit. As the MRSA reservoirs are found not only in hospital but also large reservoirs exist outside health care facilities, both the health care-associated MRSA (HA-MRSA) infections as well as the community-associated MRSA (CA-MRSA) infections, pose a threat to burn injury patients who are managed on outdoor basis with the traditional conservative dressings instead of proper surgical excision and grafting. MRSA infection usually follows carriage of the organism for some time. The “five Cs”: Contact with cases, Cleanliness, Compromised skin, Contaminated fomites, and Crowded living together with prior antibiotic exposure are all important in the epidemiology of MRSA. The cross infection among hospitalized patients can be effectively prevented by adoption of standard universal principles of contact precautions, hand hygiene and barrier nursing by all hospital workers.^29^^,^^30^ The MRSA isolates in our study were generally multi drug resistant. Except for linezolid and vancomycin to which 100% sensitivity of MRSA was found, there was variable antibiotic susceptibility pattern. Our study proved that linezolid and vancomycin were the agents that could be confidently employed on empirical basis to combat life threatening infections caused by multi drug resistant strains of MRSA. Regular periodic monitoring of the prevalence and antibiotic sensitivity of this challenging microbe is imperative. 

In our study, fungus isolates were 4.21% of the total isolates. Candidal colonization of burn wounds is more common than invasive disease and may arise from an endogenous or exogenous source. Wound colonization by fungi usually occurs later on, and *Candida spp.* are the most common non-bacterial colonizers of burn wounds and the most common cause of fungal sepsis.^31^^,^^32^ One study has reported an overall 44% fungal infection rate among 97 autopsies of burn patients where burn wounds were the commonest primary site of fungal infection and the presence of fungal elements appeared to increase over time.^33^

In our series, none of our patients developed tetanus. Tetanus may complicate the course of any trauma patient including even minor burns. We routinely instituted tetanus immunization if the last booster was over five years ago and tetanus vaccination plus anti-tetanus immunoglobulin to patients who have no history of previous vaccination. Booster vaccination is administered at 1 and 6 months for the later group.^34^^,^^35^

**Fig. 1:**
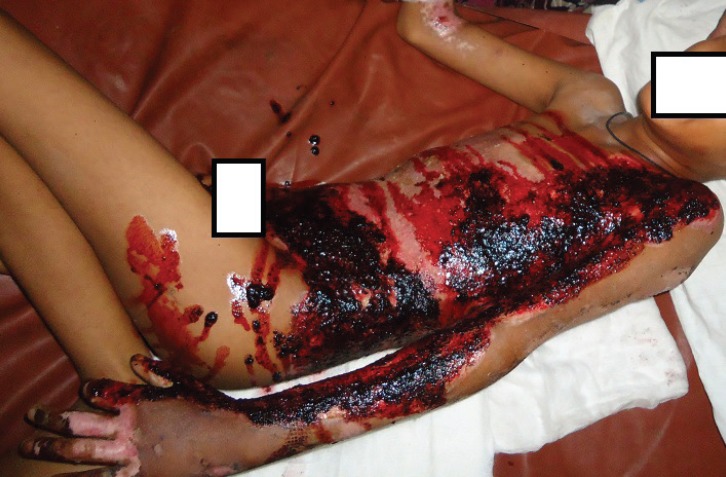
An extract from plant locally called as “*Rattanjo”* is mixed with some oil such as coconut oil and applied to the wounds by traditional healers with the belief that it will heal the wounds. These patients present typically after 2-3 weeks of failed home treatment with infected wounds needing excision and skin graft.

**Fig. 2:**
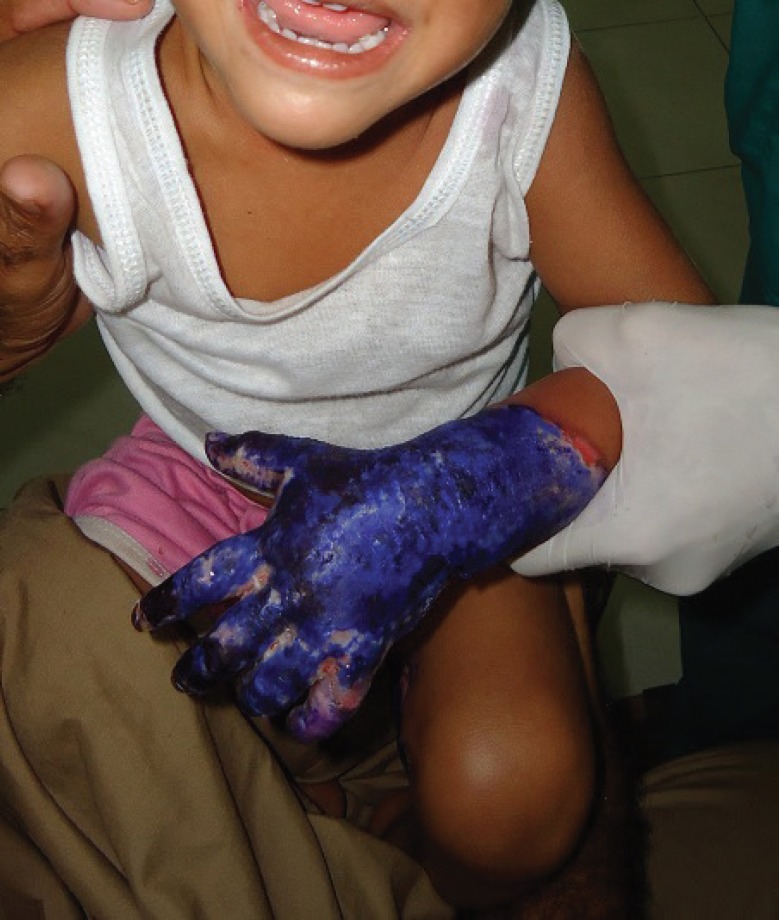
A deep burn wound initially managed at home with gentian violet by a traditional healer. These patients present at a late stage with infected wounds needing excision and skin grafting

In our study, we found a variable percentage of antibiotic resistance among the cultured organisms. Our observation concurs to several published studies.^36^^,^^37^ Not surprisingly, we found more frequent positive cultures among patients who were managed at homes for over two weeks with the traditional dressings. Most commonly the wounds of these patients were managed with application of a plant extract locally called as “*Rattanjo*” mixed in coconut oil (Figure 1). The traditional healers and quacks cover the burn wounds daily with these applications with the belief that these will heal the wounds. Additionally some of the home treated burn patients had history of their wound management with other substances such as gentian violet, and desi ghee (Figure 2). All these patients presented typically after 2-3 weeks of failed home treatment with infected wounds that needed aggressive management with wound excision and skin autografting. The published literature has also proven that the burned patients whose deep wounds are not excised and grafted early pose a greater risk of all kinds of microbial invasion, bacteraemia as well as emergence of resistant strains. In fact, early excision and grafting has become the defacto standard of care for the deep burns in today’s world.^8^^,^^23^^,^^38^^,^^39^

The present study should prompt well designed local studies to confirm and improve upon our findings. We recommend a local study to find out the relationship between wound infection and mortality among our patients. *P. aeruginosa*, *K. pneumoniae* and *S. aureus* constituted the most common bacterial microbes of burn wounds in our patients. A variable antibiotic susceptibility pattern was observed among the grown microbes. Positive cultures were significantly more frequent among patients with over two weeks duration of burn wounds. Early excision of deep burns and coverage with skin graft can help to effectively reduce the burden of these infections.

## Note

Please cite this paper as:

Saaiq M, Ahmad S, Zaib MS. Burn Wound Infections and Antibiotic Susceptibility Patterns at Pakistan Institute of Medical Sciences, Islamabad, Pakistan. World J Plast Surg 2015;4(1):9-15.

## References

[1] Mohammadi AA, Amini M, Mehrabani D, Kiani Z, Seddigh A (2008). A survey on 30 months electrical burns in Shiraz University of Medical Sciences Burn Hospital. Burns.

[2] Pasalar M, Mohammadi AA, Rajaeefard AR, Neghab M, Tolidie HR, Mehrabani D (2013). Epidemiology of burns during pregnancy in southern Iran: Effect on maternal and fetal outcomes. World Appl Sci J.

[3] Church D, Elsayed S, Reid O, Winston B, Lindsay R (2006). Burn wound infections. Clin Microbiol Rev.

[4] Raz-Pasteur A, HusseinK, Finkelstein R, UllmannY, Egozi D (2013). Blood stream infections (BSI) in severe burn patients--early and late BSI: a 9-year study. Burns.

[5] Murray C, Cunha BA (2011). Burn Wound Infections.

[6] Fitzwater J, Purdue GF, Hunt JL, O’Keefe GE (2003). The risk factors and time course of sepsis and organ dysfunction after burn trauma. J Trauma.

[7] Rafla K, Tredget EE (2011). Infection control in the burn unit. Burns.

[8] Saaiq M, Zaib S, Ahmad Sh (2012). Early excision and grafting versus delayed excision and grafting of deep thermal burns upto 40% Total body surface area: A comparison of outcome. Ann Burns Fire Disasters.

[9] Hosseini SV, Tanideh N, Kohanteb J, Ghodrati Z, Mehrabani D, Yarmohammadi H (2007). Comparison between Alpha and silver sulfadiazine ointments in treatment of Pseudomonas infections in 3rd degree burns. Int J Surg.

[10] Amini M, Kherad M, Mehrabani D, Azarpira N, Panjehshahin MR, Tanideh N (2010). Effect of Plantago major on burn wound healing in rat. J Appl Anim Res.

[11] Hazrati M, Mehrabani D, Japoni A, Montasery H, Azarpira N, Hamidian-Shirazi AR, Tanideh N (2010). Effect of honey on healing of Pseudomonas aeruginosa infected burn wounds in rat. J Appl Anim Res.

[12] Hosseini SV, Niknahad H, Fakhar N, Rezaianzadeh A, Mehrabani D (2011). The healing effect of honey, putty, vitriol and olive oil in Psudomonas areoginosa infected burns in experiental rat model. Asian J Anim Vet Adv.

[13] Tanideh N, Rokhsari P, Mehrabani D, Mohammadi Samani S, Sabet Sarvestani F, Ashraf MJ, Koohi Hosseinabadi O, Shamsian S, Ahmadi N (2014). The healing effect of licorice on Pseudomonas aeruginosa infected burn wounds in experimental rat model. World J Plast Surg.

[14] Manafi A, Kohanteb J, Mehrabani D, Japoni A, Amini M, Naghmachi M, Zaghi AH, KhaliliN (2009). Active immunization using exotoxin aconfers protection against pseudomonas aeruginosa infection in a mouse burn model. BMC J Microbiol.

[15] Japoni A, Kalani M, Farshad Sh, Ziyaeyan M, Alborzi A, Mehrabani D, Rafaatpour N (2010). Antibiotic-resistant bacteria in hospitalized patients with bloodstream infections: analysis of some associated factors. Iran Red Crescent Med J.

[16] Noorbakhsh Sabet N, Japoni A, Mehrabani D, Japoni S (2010). Multi-drug resistance bacteria in Qom hospitals, Central Iran. Iran Red Crescent Med J.

[17] Li YW, Li XX, Geng H, Tao WW, Yu CB (2013). Epidemic status of drug-resistant Mycobacterium tuberculosis in Shandong province, China. Zhonghua Jie He He Hu Xi Za Zhi.

[18] Greenhalgh DG, Saffle JR, Holmes JH 4th, Gamelli RL, Palmieri TL, Horton JW (2007). American Burn Association consensus conference to define sepsis and infection in burns. J Burn Care Res.

[19] Hussien IA, Habib KA, Jassim KA (2012). Bacterial colonization of burn wounds. J Baghdad Sci.

[20] Oncul O, Ulkur E, Acar A (2009). Prospective analysis of nosocomial infections in a burn care unit, Turkey. Indian J Med Res.

[21] Mehta M, Dutta P, Gupta V (2007). Bacterial isolates from burn wound infections and their antibiograms: A eight-year study. Indian J Plast Surg.

[22] Al Laham NA, Elmanama AA, Tayh GA (2013). Possible risk factors associated with burn wound colonization in burn units of Gaza strip hospitals, Palestine. Ann Burns Fire Disasters.

[23] Altoparlak U, Erol S, Akcay MN, Celebi F, Kadanali A (2004). The time-related changes of antimicrobial resistance patterns and predominant bacterial profiles of burn wounds and body flora of burned patients. Burns.

[24] Erol S, Altoparlak U, Akcay MN, Celebi F, Parlak M (2004). Changes of microbial flora and wound colonization in burned patients. Burns.

[25] Nasser S, Mabrouk A, Maher A (2003). Colonization of burn wounds in Ain Shams University burn unit. Burns.

[26] Srinivasan S, Vatak A, Patil A, Saldanha J (2009). Bacteriology of the burn wound at the Bai Jerbai Wadia hospital for children, Mumbai, India—A 13-year study, Part I-Bacteriological profile. Indian J Plast Surg.

[27] Kehinde AO, Ademola SA, Okesola AO, Oluwatosin OM, Bakare RA (2004). Pattern of bacterial pathogens in burn wound infections in Ibadan, Nigeria. Ann Burns Fire Disasters.

[28] Ozumba UC, Jiburum BC (2000). Bacteriology of burn wounds in Enugu, Nigeria. Burns.

[29] David MZ, Daum RS (2010). Community-associated methicillin-resistant Staphylococcus aureus: epidemiology and clinical consequences of an emerging epidemic. Clin Microbiol Rev.

[30] Gould IM (2009). Antibiotics, skin and soft tissue infection and methicillin resistant staphylococcus aureaus: cause and effect. Int J Antimicrob Agents.

[31] Schofield CM, Murray CK, Horvath EE (2007). Correlation of culture with histopathology in fungal burn wound colonization and infection. Burns.

[32] Horvath EE, Murray CK, Vaughan (2007). Fungal wound infection (not colonization) is independently associated with mortality in burn patients. Ann Surg.

[33] Murray CK, Loo FL, Hospenthal DR, Cancio LC, Jones JA, Kim SH (2008). Incidence of systemic fungal infection and related mortality following severe burns. Burns.

[34] Marshall JH, Bromberg BE, Adrizzo JR (1972). Fatal tetanus complicating a small partial thickness burn. J Trauma.

[35] Saaiq M, Zaman KU (2007). The scourge of tetanus: Time for critical re-appraisal of the issue in a broader national perspective. Ann Pak Inst Med Sci.

[36] Sader HS, Farrell DJ, Jones RN (2010). Antimicrobial susceptibility of gram-positive cocci isolated from skin and skin structure infections in European medical centres. Int J Antimicrob Agents.

[37] Guggenheim M, Zbinden R, Handschin AE, Gohritz A, Altintas MA, Giovanoli P (2009). Changes in bacterial isolates from burn wounds and their antibiograms: a. 20-year study (1986–2005). Burns.

[38] Ekrami A, Kalantar E (2007). Analysis of the bacterial infections in burn patients at Taleghani burn hospital in Ahvaz, Khuzestan province. Iranian J Clin Dis.

[39] Ong YS, Samuel M, Song C (2006). Meta-analysis of early excision of burns. Burns.

